# 24th International Conference on Safe Community: count down to decade of action for road safety

**DOI:** 10.5249/jivr.v12i3.1372

**Published:** 2020-08

**Authors:** Dale Hanson, Saeed Namaki, Mohammad Hossein Somi, Homayoun Sadeghi Bazargani, Ray Shuey, Rahim Farahnak Benekohal, Michael Lowery Wilson, Guenter Lob, Reza Mohammadi, Reza Deljavan Anvary, Mohammad Saadati

**Affiliations:** ^*a*^ Associate Professor, Chair, International Safe Community Certifying Center, Australia.; ^*b*^ Minister of Health and Medical Education, Tehran, Iran.; ^*c*^ Liver and Gastrointestinal Diseases Research Center, Chancellor, Tabriz University of Medical Sciences, Tabriz, Iran.; ^*d*^ Road Traffic Injury Research Center, Tabriz University of Medical Sciences, Tabriz, Iran.; ^*e*^ Principal and Founder, Strategic Safety Solutions Pty Ltd. Australia.; ^*f*^ Professor of Civil and Environmental Engineering, University of Illinois at Urbana-Champaign, Urbana, USA.; ^*g*^ Injury Epidemiology and Prevention Research Group, Turku Brain Injury Center, Division of Clinical Neurosciences, University Hospital and University of Turku, Turku, Finland.; ^*h*^ Department of Trauma Surgery, Ludwig Maaximillians-University Munich, Munich, Germany.; ^*i*^ Public Health Department, Karolinska Institute, Stockholm, Sweden.; ^*j*^ Neuroscience research center, Road Traffic Injury Research Center, Tabriz University of Medical Sciences, Tabriz, Iran.

Worldwide, every year 1.35 million people die due to road traffic injuries (Global Traffic Safety Report 2018). Road traffic injuries are now the leading cause of death for children and young people (5-29 years old), as re-ported by the World Health Organisation (WHO). 

The ‘Decade of Action’ for road safety (2011-2020) was a universal start to save millions of lives by taking a multi-disciplinary collaboration approach for road safety management. It encouraged countries to have safe road infrastructures, safe vehicles, safety-enhanced user behavior and improved road-injury related health services. All countries were encouraged to join this movement to achieve Sustainable Development Goals (SDGs).

The ‘Safe Community’ program is a framework to employ local resources and public participation to improve the community safety and the empowerment of the population which will lead to SDGs. So, there is great potential for local communities to improve the safety of their people.

Now, in the last year of the Decade of Action for road safety, we have an opportunity to review our achievements, strengths and weaknesses, and future needs so we can develop an action plan for the second decade that will be more equitable and evidence-based considering the local characteristics. 

The 24th International Conference on Safe Community gathers us to discuss and share our experiences and scientific achievements. Sharing and learning from other safe communities about road safety improvements provides a great example for local communities to employ a bottom-up approach to prevent road trauma due to road traffic injuries. 

